# DNA methylation in peripheral tissue of schizophrenia and bipolar disorder: a systematic review

**DOI:** 10.1186/s12863-016-0332-2

**Published:** 2016-01-25

**Authors:** Nina Teroganova, Leah Girshkin, Catherine M. Suter, Melissa J. Green

**Affiliations:** School of Psychiatry, University of New South Wales, Randwick, NSW Australia; Schizophrenia Research Institute, 405 Liverpool St, Darlinghurst, NSW 2010 Australia; Molecular Structural and Computational Biology Division, Victor Chang Cardiac Research Institute, Darlinghurst, NSW 2010 Australia; Neuroscience Research Australia, Sydney, NSW 2031 Australia

**Keywords:** Epigenetics, Psychosis, Mood disorder, RELN, COMT, BDNF

## Abstract

**Background:**

Increasing evidence suggests the involvement of epigenetic processes in the development of schizophrenia and bipolar disorder, and recent reviews have focused on findings in post-mortem brain tissue. A systematic review was conducted to synthesise and evaluate the quality of available evidence for epigenetic modifications (specifically DNA methylation) in peripheral blood and saliva samples of schizophrenia and bipolar disorder patients in comparison to healthy controls.

**Methods:**

Original research articles using humans were identified using electronic databases. There were 33 included studies for which data were extracted and graded in duplicate on 22 items of the Strengthening the Reporting of Observational Studies in Epidemiology (STROBE) statement, to assess methodological precision and quality of reporting.

**Results:**

There were 15 genome-wide and 18 exclusive candidate gene loci investigations for DNA methylation studies. A number of common genes were identified as differentially methylated in schizophrenia/bipolar disorder, which were related to reelin, brain-derived neurotrophic factor, dopamine (including the catechol-O-methyltransferase gene), serotonin and glutamate, despite inconsistent findings of hyper-, hypo-, or lack of methylation at these and other loci. The mean STROBE score of 59 % suggested moderate quality of available evidence; however, wide methodological variability contributed to a lack of consistency in the way methylation levels were quantified, such that meta-analysis of the results was not possible.

**Conclusions:**

Moderate quality of available evidence shows some convergence of differential methylation at some common genetic loci in schizophrenia and bipolar disorder, despite wide variation in methodology and reporting across studies. Improvement in the clarity of reporting clinical and other potential confounds would be useful in future studies of epigenetic processes in the context of exposure to environmental and other risk factors.

## Background

Schizophrenia (SZ) and bipolar disorder (BD) share some common genetic vulnerability [1, 2] and environmental risk factors [1, 3]. Only a small portion (approximately 23 %) of the variance in risk for these disorders can be accounted for by common variation in the genome [4, 5]. The role of epigenetic processes (affecting gene *expression*) may thus account for substantial variation in the development of SZ and BD [6], and is consistent with evidence for non-genetic risk factors (e.g., obstetric complications [7, 8] and viral infections [9]) which may confer risk for these disorders via epigenetic processes.

Epigenetic modifications to the genome refer to changes in the physical structure of the chromatin, without a change in the DNA sequence itself [10]. The most widely studied epigenetic modification is DNA methylation, characterised by covalent linking of a methyl (CH3) group to a cytosine residue [11], almost exclusively occurring at cytosines within CpG dinucleotides. These CpGs are clustered in ‘CpG islands’ that tend to be located in regulatory elements of the gene, such as promoters or enhancers [12]. Methylation at CpG islands usually results in transcriptional silence of the associated gene [12]. In recent studies of psychiatric phenomena, the functional impact of stress-related hypomethylation of genetic loci known to regulate stress responses (e.g., FK506 binding protein 5 *(FKBP5*)) suggest that this process may be relevant to many stress-related disorders [13]. While there are a number of post-mortem studies reporting differentially methylated genes in these disorders, these findings have been recently reviewed elsewhere [14, 15]. This review instead focused on the growing evidence base for differential DNA methylation in peripheral (i.e. blood and saliva) samples, which may minimise confounding effects related to tissue quality and stability [16], and importantly allow the study of epigenetic processes in living humans. Recent comparison of within-subject methylation patterns across blood and brain suggest the utility of peripheral blood in human epigenetic studies [17].

With the increasing use of peripheral tissue for the study of methylation patterns in psychotic disorders, the aim of this study was to perform a systematic review of evidence from observational case–control studies investigating differential DNA methylation in the peripheral tissues (blood or saliva) of SZ and/or BD patients, in comparison to a healthy control (HC) group. Assessment of the quality, consistency and strength of evidence reported across studies was undertaken for all studies using accepted criteria, using a validated tool for assessing methodological precision and quality of reporting.

## Methods

### Literature search: inclusion/exclusion criteria

Included are peer-reviewed, observational case–control studies investigating DNA methylation in the peripheral tissues (blood, saliva) of SZ (including schizoaffective disorder) and/or BD (type I and II) in comparison to a HC group. Excluded studies reported other types of epigenetic modifications (i.e. hydroxymethylation), mRNA gene products of the methylation pathway, or DNA methylation in germ line cells or post-mortem brain tissue, for which results have recently been reviewed elsewhere [14, 15].

### Search strategy

Systematic searching of electronic databases MEDLINE, EMBASE, PsychINFO and PubMed identified studies published between 2000 and February 2015; further hand searching was conducted until April 2015. The following key terms were used: exp schizophrenia/, schizophreni$.tw, schizo$.tw, exp bipolar disorder/, bipolar disorder.tw, exp psychosis/, psychosis.tw, dna methylat$.tw, demethylat$.tw, hypomethylat$.tw and hypermethylat$.tw. Searches were limited to studies published in English, conducted in humans, and published after the year 2000 to minimise the methodological inconsistencies seen in the earliest studies of DNA methylation (e.g. improvements in polymerase chain reaction based DNA methylation methods) [18].

### Study selection

**Fig. 1:**
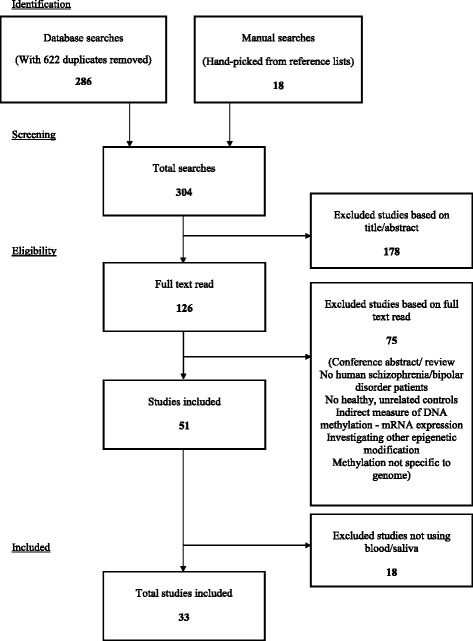
PRISMA flowchart - systematic selection process for included and excluded studies of this review

A preferred reporting items for systematic reviews and meta-analysis (PRISMA) flowchart of the search and selection processes of the included studies is presented in Fig. 1. All decisions relating to study inclusion were completed independently by two authors (NT and LG) with any disagreements resolved by discussion with MG.

### Quality assessment and study characteristics

Information relating to data quality were graded in duplicate (authors NT and MG) using 22 items listed in the Strengthening the Reporting of Observational Studies in Epidemiology (STROBE) statement [19], to assess the risk of bias within studies and across studies, methodological consistency and precision, as well as reporting transparency and comprehensibility. The STROBE statement consists of a 22 item checklist which allows a systematic and critical assessment of the strengths and weakness of the study design, conduct and analysis [19]. Thresholds for determining study quality were determined by authors, as per the STROBE guidelines: scores on the STROBE checklist of ≥ 66 % were considered to be reflective of high study quality, ≤ 33 % of low quality reporting, and scores in between this range were of moderate quality. Thresholds for categorising significant change in methylation status for hypermethylation, hypomethylation and differential methylation (i.e. non-specified direction of difference), as well as no significant group difference in methylation were protocol dependent, but largely reflect convention of significance level being set at 0.05, with appropriate corrections applied according to the number of analyses being conducted. Study characteristics are summarised in Tables 1, 2 and 3 and include sample characteristics, methods of quantifying DNA methylation, as well as any relevant data (e.g., medication status) that could contribute to the investigation of subgroups.

**Table 1:** Summary of studies reporting DNA methylation in schizophrenia

Ref.	N		Mean age (SD)		Sex (F %)		Method	Subgroup analyses /covariates	RESULTS: methylation loci
	SZ	HC	SZ	HC	SZ	HC			
Aberg *et al.,* 2014 [20]	759	738	53 (12)	55 (12)	45	46	MBD protein-enriched genome sequencing ^b, d, e^	Age, sex, smoking ^m^, alcohol use ^m^, medication ^m^, autoimmune disorders ^m^	Differential methylation ^l^ of FAM63B, RELN (first intron), FCAR and 8 other genes linked to hypoxia & the immune system
Aberg *et al.,* 2012 [21]	750	750	-	-	-	-	MBD protein-enriched genome sequencing ^b, d, i^	Age, sex	Differential methylation^l^ of GRIA2, HTRA3, CAMK2D, FNDC3B and DCTN
Bonsch *et al.,* 2012 [22]	27	34	-	-	30	47	Modified non-radioactive elongation assay and MSRE-quantitative PCR ^a, c, d, e^	Sex ^k^, medication ^j^, promoter methylation compared to global DNA methylation	Global methylation differences ^l^; Lower methylation of RELN and SOX10 promoters ^c, m^ in SZ; SZ on medication had similar methylation levels to HC
Bromberg *et al.,* [23]	28	26	39 (14)	42 (10)	64	62	Radiolabelled [3H] cytosine-extension assay ^a, d, e^	Age ^m^, sex, smoking ^l^, illness duration ^m^, medication ^m^	No global methylation differences ^m^; Higher methylation in SZ non-smokers
Chen *et al.,* 2012 [24]	371	288	-	-	46	57	Bisulfite sequencing ^c, d, e^	Sex ^j^	No differential methylation of MAOA (promoter) ^m^ Greater methylation in SZ females compared to males
Ikegame *et al.,* 2013 [25]	100	100	43 (13)	46 (12)	46	45	Pyrosequencing ^c, d, e^	Age ^m^, sex ^j^	Hypermethylation of BDNF (promoter I) ^j^ for SZ CpG-72 compared HC, however methylation generally low in SZ/HC; No diffferential methylation of BDNF (promoter IV) ^m^ between groups; higher methylation in SZ/HC females at all CpG sites
Kinoshita *et al.,* 2014 [26]	63	42	49 (10)	47 (10)	22	40	450 K methylation array ^a, d, f^	Age, sex, cell type heterogeneity ^l^	Global methylation differences (485 764 CpG sites) ^l^ Hypermethylation in SZ found in 1161 CpG sites when controlling for cellular heterogeniety
Kinoshita *et al.,* 2013a [27]	42	42	52 (7)	52 (6)	0	0	450 K methylation array ^a, d, e^	Age, medication	Global methylation differences (164 657 CpG sites) ^l^ including SLC18A2, GNAL, KCNH2 and NTNG2
Kinoshita *et al.,* 2013b [28]	24	23	31 (11)	31 (10)	54	57	450 K methylation array ^a, d, e^	Sex ^l^	Global methylation differences in SZ (485 764 CpG sites) ^l^ including B3GAT2, HDAC4, DGKI, PCM1, INSIG2, GFRA2 and RAI1; Did not replicate published methylation findings in SZ for COMT, HTA1A and MAOA
Kordi-Tamandani *et al.,* 2013a [29]	81	71	48 (11)	47 (12)	25	20	Methylation specific PCR ^c, d, g^	-	Hypermethylation of GMR2 ^j^, GMR5 ^j^, GRIA3 ^j^, GMR8 ^j^ (all promoter regions)
Kordi-Tamandani *et al.,* 2012 [30]	80	71	48 (11)	47 (12)	-	-	Methylation specific PCR ^c, d, g^	-	Differential methylation of BDNF (promoter) ^l^ and DAT1 ^j^
Kordi-Tamandani *et al.,* 2013b [31]	94	99	48 (11)	47 (12)	29	27	Methylation specific PCR ^c, d, g^	Genotype ^m^	Hypermethylation of CTLA4 (promoter) ^j^ increased in SZ
Liao *et al.,* 2014 [32]	2	1	25 (4)	31 (0)	100	100	MBD protein-enriched genome sequencing ^a, c, d, h^	Paranoid ^j^/undifferentiated illness type ^l^	Global methylation differences ^l^; GRB2 ^j^, PRKCA ^j^, DLG4 ^j^, MAPT-S1 ^j^, DISC1 ^j^ and 16 other genes; Differential methylation mostly found in intergenic and intronic regions
Liu *et al.,* 2013 [33]	98	108	34 (11)	-	25	36	27 K methylation assay ^a, d, f^	Age, sex, ethnicity, alcohol/nicotine/cannabis use, SZ symptoms ^l^, medication ^j^, illness duration ^j^, age of onset ^l^	Global methylation differences (7562 CpG sites) in SZ ^l^: MS4A1 ^j^, MPG ^k^, SLC25A10 ^j^, CBFA2T3 ^k^ and 17 other genes ^l^ linked to inflammatory response, haematological development and cytotoxic reactions; Hypermethylation of MS4A1 associated with chlorpromazine dosage; Higher methylation of MPG and SLC25A10 associated with longer illness duration; Hypomethylation of CBFA2T3 associated with age of SZ onset
Melas *et al.,* 2012 [34]	177	171	52 (9)	-	51	-	Luminometric methylation assay and bisulfite sequencing ^a, c, d, e^	Age ^m^, sex ^m^, smoking ^m^, alcohol use ^m^, medication (haloperidol) ^l^, hospital admissions ^m^, length of hospital stay ^m^, familial absence of SZ ^m^, age of onset ^k^	Global methylation differences in SZ ^l^ ; No differential methylation of 5-HTT (8 CpG sites) ^m^; hypermethylation of S-COMT (5 CpG sites) ^j^
Murphy *et al.,* 2008 [35]	18	31	-	-	-	-	Bisulfite sequencing ^c, d, e^	-	No differential methylation of SYNIII ^m^
Murphy *et al.,* 2005 [36]	20	31	-	-	-	-	Bisulfite sequencing ^c, d, i^	-	No differential methylation of S-COMT (promoter) ^m^
Nishioka *et al.,* 2013 [37]	17	15	23 (5)	23 (4)	59	33	27 K methylation assay ^a, d, f^	Sex ^m^, SZ symptoms, GAF score, duration of untreated psychosis, age at onset	Global methylation differences (603 CpG sites) in SZ ^l^ : COMTD1 (promoter) ^l^, SLC6A3 ^k^, HTR1E ^l^ and 7 other genes ^l^ related to the nuclear lumen, transcription factor binding and nucleotide binding
Ota *et al.,* 2014 [38]	51	51	25 (8)	26 (8)	37	37	Bisulfite sequencing ^c, d, i^	Age ^m^, sex ^j^	Hypermethylation of GCH1 ^j^ (CpG13, CpG15, CpG16 and CpG21 only) in first episode psychosis; Only CpG21 ^j^ in SZ males
Pun *et al.,* 2011 [39]	30	30	-	-	50	37	Bisulfite sequencing ^c, d, e^	Single-nucleotide polymorphism	Hypermethylation of GABRB2 in SZ (CpG sites 1–26) ^j^
Shimabukuro *et al.,* 2007 [40]	210	237	-	-	41	54	HPLC ^a, d, f^	Age ^l^, sex ^k^, subtypes of SZ ^m^	Global hypomethylation in SZ ^k^ In SZ males hypomethylation decreases with age
Van Eijk *et al.,* 2014 [41]	264	252	-	-	-	-	27 K methylation assay ^a, d, f^	Age, sex, gene expression associations ^l^	Global methylation differences (11 320 CpG sites)in SZ ^l^: including PRRT1, HLA-C, MRPL41, CALHM1; Significant association between DNA methylation and gene expression

*SZ* schizophrenia, *HC* healthy control, *N* sample number, *F* female, *SD* standard deviation, *MBD* methyl-CpG-binding domain, *MSRE* methylation specific restriction enzymes, *HPLC* high performance liquid chromatography, *UTR* untranslated regions, *PCR* polymerase chain reaction, *GAF* global assessment of functioning. Genes: *FAM63B* family with sequence similarity 63 member B, *RELN* reelin, *FCAR* Fc fragment of IgA receptor, *GRIA2* glutamate receptor ionotropic alpha-amino-3-hydroxy-5-methyl-4-isoxazole propionic acid 2, *HTRA3* HtrA serine peptidase 3, *CAMK2D* calcium/calmodulin-dependent protein kinase 2 delta, *FNDC3B* fibronection type 3 domain containing 3B, *DCTN* dynactin, *SOX10* sex determining region Y box 10, *MAOA* monoamine oxidase A, *BDNF* brain-derived neurotrophic factor, *SLC18A2* vesicular monoamine transporter 2, *GNAL* guanine nucleotide binding protein G alpha activating polypeptide olfactory type, *KCNH2* potassium voltage-gated channel subfamily H member 2, *NTNG2* netrin G2, *B3GAT2* beta-1 3-glucuronyltransferase 2, *HDAC4* histone deacetylase 4, *DGKI* diacylglycerol kinase iota, *PCM1* pericentriolar material 1, *INSIG2* insulin induced gene 2, *GFRA2* glial cell line-derived neurotrophic factor family receptor alpha 2, RAI1, retinoic acid induced 1, *GMR2* glutamate metabotrophic receptor 2, *GMR5* glutamate metabotrophic receptor 5, *GRIA3* glutamate receptor ionotrophic alpha-amino-3-hydroxy-5-methyl-4-isoxazole propionic acid 3, *GMR8* glutamate metabotrophic receptor 8, *DAT1* dopamine active transporter 1, *CTLA4* cytotoxic T-lymphocyte-associated protein 4, *GRB2* growth factor receptor-bound protein 2, *PRKCA* protein kinase C alpha, *DLG4* discks large homolog 4, *MAPT-S1* microtubule-associated protein tau, *DISC1* disrupted in schizophrenia 1 protein, *GCH1* guanosine triphosphate cyclohydrolase 1, *PRRT1* proline-rich transmembrane protein 1, *HLA-C* human leukocyte antigen receptor C, *MRPL41* mitochondrial ribosomal protein L41, *MS4A1* membrane-spanning 4-domains subfamily A member 1, *MPG* N-methylpurine-DNA glycosylase, *SLC25A10* solute carrier family 25 member 10, *CBFA2T3* core-binding factor alpha subunit 2 translocated to 3, *5-HTT* serotonin neurotransmitter transporter, *SYNIII* synapsin 3, *S-COMT* soluble catechol-O-methyltransferase, *COMTD1* catechol-O-methyltransferase domain containing 1, *SLC6A3* solute carrier family 6 transporter member 3, *HTR1E* 5-hydroxytryptamine serotonin receptor 1E G protein-coupled, *GABRB2* gamma-aminobutyric acid A receptor beta 2

Study type

^a^ global DNA methylation

^b^ methylome-wide association study

^c^ candidate gene study

Tissue type

^d^ blood

Measure of Methylation

^e^ percentage

^f^ beta-value

^g^ odds ratio

^h^ peak score

^i^ no reported

Results

^j^ significant hypermethylation

^k^ significant hypomethylation

^l^ significant differentially methylated

^m^ no significant difference

**Table 2:** Summary of studies reporting DNA methylation in bipolar disorder

Ref.	N		Mean age (SD)		Sex (F %)		Method	Subgroup analyses /covariates	RESULTS: methylation loci
	BD	HC	BD	HC	BD	HC			
Bromberg *et al.,* 2009 [42]	49	27	39 (13)	42 (10)	41	37	Radiolabeled [3H] cytosine-extension assay ^a, c, d^	Medication (valproate) ^h^, sex ^h^, smoking ^h^, duration of illness ^h^, family history of BD ^h^	No global methylation differences ^h^
Carlberg *et al.,* 2014 [43]	60	278	42 (15)	32 (4)	45	62	MethyLight ^b, c, d^	Age ^g^, gender ^h^, clinical variables, genotype ^h^	No difference in methylation of BDNF (exon I promoter) ^h^
D’Addario *et al.,* 2012 [44]	94	52	52 (12)	-	60	-	Fluorescence-based real-time PCR ^b, c, d^	Medication ^e^, BD-I compared to BD-II ^h^, mood state ^h^	Hypermethylation of BDNF (exon I promoter) ^e^ only in BD-II; Higher methylation associated with mood stabiliser and antidepressants, but lower for lithium and valproate
Dell’Osso *et al.,* 2014 [45]	111	44	-	-	-	-	Fluorescence-based real-time PCR ^b, c, d^	Age ^h^, sex ^h^, mood state ^e^, medication, BD-I compared to BD-II	Hypermethylation of BDNF (exon I promoter) ^e^ in BD-II compared to BD-I; Higher methylation in depressed compared to manic/mixed states; Higher methylation in BD-II males with increasing age
Kaminsky *et al.,* 2012 [46]	370	382	43 (11)	42 (6)	58	55	Pyrosequencing ^b, c, d^	Age ^e^, sex, genotype ^f^, medication (mood stabiliser) ^e^	HCG9 (first exon extending into the first intron) ^f^ in BD (when controlling for age and genotype); Higher methylation in BD/HC with increasing age; Lower methylation in GG allele compared to GA allele carriers; Higher mood stabiliser dose increases methylation towards HC levels
Kuratomi *et al.,* 2008 [47]	23	18	57 (11)	46 (12)	52	33	Pyrosequencing ^b, c, d^	Age ^h^, sex ^e^, medication (valproate) ^h^, BD-I compared to BD-II ^f^	Differential methylation of SMS (5'region) ^g^, higher methylation for females in BD-I/II group compared to HC; Hypomethylation of PPIEL (promoter and 5'region) ^f^ for BD-II compared to BD-I; No difference in methylation of PIP5KL1 ^h^, ARMC3 ^h^
Sugawara *et al.,* 2011 [48]	20	20	39 (13)	39 (9)	60	20	Pyrosequencing ^b, c, d^	-	Differential methylation of SLC6A4 (promoter) ^g^, higher methylation in CpG 3 and 4 for BD compared to HC

*BD* bipolar disorder, *HC* healthy control, *N* sample number, *F* female, *SD* standard deviation, *PCR*, polymerase chain reaction. Genes: *BDNF* brain-derived neurotrophic factor, *HCG9* human leukocyte antigen complex group 9, *SMS* spermine synthase, *PPIEL* peptidylprolyl isomerase E-like, *PIP5KL1* phosphatidylinositol-4-phosphate 5-kinase-like 1, *ARMC3* armadillo repeat containing 3, *SLC6A4* serotonin transporter solute carrier family 6 member 4

Study type

^a^ global DNA methylation

^b^ candidate gene study

Tissue type

^c^ blood

Measure of Methylation

^d^ percentage

Results

^e^ significant hypermethylation

^f^ significant hypomethylation

^g^ significant differentially methylated

^h^ no significant differences

**Table 3:** Summary of studies reporting DNA methylation in bipolar disorder and schizophrenia in the same study

Ref.	N			Mean age (SD)			Sex (F %)			Method	Subgroup analyses/covariates	RESULTS: methylation loci
	SZ	BD	HC	SZ	BD	HC	SZ	BD	HC			
Carrard *et al.,* 2011 [49]	40	58	67	32 (8)	42 (10)	42 (12)	40	57	27	HRM assay ^b, c, e^	Age ^j^, sex ^j^, symptoms ^j^	5-HTR1A ^g^ for SZ/BD compared to HC and for SZ compared to BD
Gradirivasf *et al.,* 2011 [50]	24	24	24	-	-	-	-	-	-	Bisulfite sequencing and qMSP ^b, d, e^	Age ^h^, sex ^j^, genotype ^h^, medication (antipsychotics) ^h^, marital status ^j^, smoking ^j^, alcohol abuse ^j^, education ^j^	No differential methylation of HTR2A (promoter) ^j^ for most CpG sites except for 1438A/G, 1420 and 1223 polymorphic sites; Hypomethylation of T120C site in SZ/BD ^h^; Lower methylation in SZ CC allele carriers with increasing age
Li *et al.,* 2014 [51]	6	3	1	24 (7)	47 (11)	-	67	33	-	Methylated DNA immunoprecipitation ^a, c, f^	Age ^i^, sex ^i^	Hypermethylation of ADRB1 ^g^, HTR1A ^g^, NPAS1 ^g^ and hypomethylation of COMT ^h^ in SZ; HNRNPA1 and 56 other genes differentially methylated in both SZ & BD ^i^; 11 genes were differentially methylated among SZ & BD ^i^
Nohesara *et al.,* 2011 [52]	20	20	25	-	-	-	-	-	-	Bisulfite sequencing and qMSP ^b, d, e^	Age ^g^, sex, marital status ^j^, genotype ^j^	Hypomethylation of MB-COMT (promoter) ^h^ Higher methylation in SZ with increasing age

*SZ* schizophrenia; *BD* bipolar disorder; *HC* healthy control; *N* sample number; *F* female; *SD* standard deviation; *HRM* high resolution melt; *qMSP* quantitative methylation specific polymerase chain reaction. Genes: *5-HTR1A* serotonin 1A receptor; *HTRA2A* serotonin 2A receptor; *ADRB1* adrenoreceptor beta 1; *NPAS1* neuronal PAS domain-containing protein 1; *HNRNPA1* heterogenous nuclear ribonucleoprotein A1; *MB-COMT* membrane-bound catechol-O-methyltransferase

Study type

^a^ methylome-wide association study

^b^ candidate gene study

Tissue type

^c^ blood

^d^ saliva

Measure of Methylation

^e^ percentage

^f^ peak score

Results

^g^ significant hypermethylation

^h^ significant hypomethylation

i significant differentially methylated

^j^ no significant differences

## Results

### Search results: included and excluded studies

The systematic search strategy identified a total of 908 publications, of which 622 were duplicates (i.e., 286 unique studies); an additional 18 publications were found by hand searching reference lists and advance access publications (See Fig. 1). These 304 studies were screened for relevance by title and abstract, resulting in the removal of 178 studies. Full text screening of the remaining 126 studies excluded a further 75 studies which did not meet inclusion criteria (see Fig. 1); of these, 41 were conference abstracts/reviews, 22 did not include SZ or BD participants, two did not have a comparison group consisting of healthy unrelated subjects, seven reported indirect measures of DNA methylation (i.e. mRNA expression of DNA methylation products), two investigated other types of epigenetic modification and one study did not investigate DNA methylation in the genome. An additional 18 studies were excluded which conducted DNA methylation analyses using only germ line cells or post-mortem tissue. A final total of 33 studies, which fulfilled inclusion criteria, were evaluated in this systematic review.

### Study quality assessment

The STROBE ratings suggested that the available evidence for differential methylation in SZ and BD ranged from low (29.5 % minimum) to high quality (77 % maximum) with the mean of all scores at 59 % (SD: 2.36), suggesting moderate quality of available evidence and moderate probability of reporting bias.

### Sample characteristics

The 33 included studies examining differential DNA methylation in peripheral tissues comprised 22 studies that compared SZ to HC [20–41] (see Table 1), seven studies that compared BD to HC [42–48] (with three studies also comparing BD-I to BD-II; see Table 2), and four studies that compared HC to both SZ and BD [49–52] (SZ/BD; see Table 3). The most common tissue for methylation was blood (n = 31; SZ: 22, BD: 7, SZ/BD: 2), however two studies reported the use of saliva (both were SZ/BD studies). Tables 1, 2 and 3 summarise sample characteristics for the 33 included studies. Sample sizes varied considerably across studies (for SZ, M = 130.6; SD = 203.4; range = 2-759; for BD, M = 75.6, SD = 103.2, range = 3-370; for HC, M = 125.3; SD = 185.7; range = 1-750; see Tables 1, 2 and 3) with the mean age being 39.1 years (SD = 11.3, range = 23-53 years) for SZ, 45.3 years (SD = 7.4; range = 39-57 years) for BD, and 40.9 years (SD = 8.9; range = 23-12 years) for HC (see Tables 1, 2 and 3). The mean percentage of females per sample was 43.7 % for SZ, 50.8 % for BD and 42.4 % for HC.

### Methodological variability

There were 16 different methods reported in the 33 included studies, with four studies using more than one method to determine methylation status. The most commonly used methodology for candidate gene loci was bisulfite sequencing of candidate genetic loci (n = 8) [24, 34–36, 38, 39, 50, 52], while the most commonly reported genome-wide methods used were methyl-CpG-binding domain (MBD) protein-enriched genome sequencing (n = 3) [20, 21, 32], 450 K arrays (n = 3) [26–28], and 27 K arrays (n = 3) [33, 37, 41]. Other methods for the study of candidate genetic loci were pyrosequencing (n = 4) [25, 46–48], methylation specific polymerase chain reaction (PCR; n = 3) [29–31], fluorescence-based real-time PCR (n = 2) [44, 45], quantitative methylation specific PCR (n = 2) [50, 52], methylation sensitive restriction enzyme (MSRE) quantitative PCR (n = 1) [22], MethyLight protocol (n = 1) [43] and high-resolution melt (HRM) method (n = 1) [49]. Other methods used to measure genome-wide DNA methylation were radiolabelled [3H] cytosine-extension assay (n = 2) [23, 42]; modified non-radioactive elongation assay (n = 1) [22], luminometric methylation assay (n = 1) [34], high-performance liquid chromatography (HPLC; n = 1) [40] and methylated DNA immunoprecipitation (n = 1) [33]. These inconsistencies in the way that methylation was quantified precluded meta-analysis.

### Methylation analyses and genes investigated in schizophrenia and bipolar disorder

Genome-wide DNA methylation analyses (including three methylome-wide association study; MWAS) were conducted in 15 out of 33 studies (comprising 13 SZ studies, one BD sample, and one combined SZ/BD sample). Two of these 15 genome-wide DNA methylation studies (one SZ and one BD) reported no difference in DNA methylation status between clinical cases and controls [23, 42], while one study found genome-wide hypomethylation in SZ [40]. Of the 15 genome-wide studies, only four reported estimates of ‘global’ methylation changes across the entire genome (i.e., % differential methylation without reference to specific genes). The remaining 18 studies focused exclusively on candidate gene loci (9 SZ, 6 BD and 3 SZ/BD studies). There was a total 163 different genes investigated, with four genes investigated in more than one study. These included reelin *(RELN)* (2 SZ studies), brain-derived neurotrophic factor *(BDNF)* (3 SZ and 3 BD studies), catechol-O-methyltransferase *(COMT)* (1 SZ/BD, 3 SZ studies) and hydroxytryptamine serotonin 1A receptor *(HTR1A)* (2 SZ/BD studies). Out of the 33 studies, 3 provided a raw results database for download [32, 41, 51].

### Evidence for DNA methylation in schizophrenia and bipolar disorder

Across all studies of SZ and/or BD, there were 21 sites reported as hypermethylated, seven sites of hypomethylation, and 135 genetic loci reported as differentially methylation. The most common genes identified as differentially methylated in SZ/BD were different receptors, transporters and neurotransmitters related to *RELN*, *BDNF*, dopamine, serotonin and glutamate (see Table 4); this consisted of 14 candidate gene loci studies (one *RELN*, five *BDNF*, five dopamine, two serotonin and one glutamate) and 10 genome-wide studies (one *RELN*, one *BDNF*, three dopamine, four serotonin and one glutamate). For these genes, there was evidence of both hyper- and hypo- methylation in both SZ and BD, as well as some evidence for lack of differential methylation. There were also several studies reporting DNA methylation of genes previously linked to SZ, including: hypermethylation of gamma-aminobutyric acid receptor beta-2 (*GABRB2*) [39], discs large homolog 4 (*DLG4*) and the gene disrupted in schizophrenia 1 (*DISC1)* [32], as well as differential methylation of major histocompatibility complex class C (*HLA-C)* and calcium homeostatis modulator 1 *(CALHM1)* [41]. The results of specific genetic loci reported in more than one study are discussed in further detail below. In addition, 11 studies (7 SZ, 3 BD and 1 SZ/BD study) reported no differences in methylation in a number of genes (see Tables 1, 2 and 3).

**Table 4:** Most commonly identified differentially methylated genes and related systems in schizophrenia and bipolar disorder studies

Summary		Genetic loci	Results					Gene expression status	Group
Loci	References		Candidate loci			Global DNA			
Serotonin	[49]	5-HTR1A	^a^					-	SZ, BD
	[51]	HTR1A				^c, a^		Decrease (in SZ)	SZ, BD
	[50]	HTR2A			^d^			Increase (SZ & BD)	SZ, BD
	[48]	SLC6A4				^c^		Decrease (S/S genotype only)	BD
	[37]	HTR1E				^c^		-	SZ
	[34]	5-HTT					^d^	-	SZ
Glutamate	[21]	GRIA2				^c^		-	SZ
	[29]	GMR2 GMR5 GMR8 GRIA3	^a^					Increase (GRM2, GRM5 & GRIA3 only)	SZ
BDNF	[44]	BDNF exon 1 promoter	^a^					-	BD-II
	[45]		^a^					Decrease (BD-II only)	BD-II
	[43]				^d^			-	BD
	[25]	BDNF promoter I	^a^					-	SZ
	[30]	BDNF promoter				^c^		Increase	SZ
	[25]	BDNF promoter IV			^d^			-	SZ
Dopamine	[30]	DAT1	^a^					No difference	SZ
	[37]	SLC6A3		^b^				-	SZ
	[27]	SLC18A2				^c^		-	SZ
*COMT*	[52]	MB-COMT		^b^				Increase (SZ & BD)	SZ, BD
	[34]	S-COMT	^a^					-	SZ
	[51]	COMT				^c, b^		-	SZ, BD
	[37]	COMTD1 promoter				^c^		-	SZ
	[36]	S-COMT promoter			^d^			-	SZ
RELN	[20]	RELN intron 1				^c^		-	SZ
	[21]	RELN promoter			^d^			-	SZ

*SZ* schizophrenia; *BD* bipolar disorder; *5-HTR1A* 5-hydroxytryptamine serotonin 1A receptor; *HTR2A* 5-hydroxytryptamine serotonin 2A receptor; *SLC6A4* serotonin transporter solute carrier family 6 member 4; *HTR1E* 5-hydroxytryptamine serotonin receptor 1E G protein-coupled; *5-HTT* serotonin neurotransmitter transporter; *GRIA2* glutamate receptor ionotropic alpha-amino-3-hydroxy-5-methyl-4-isoxazole propionic acid 2; *GMR2* glutamate metabotrophic receptor 2; *GMR5* glutamate metabotrophic receptor 5; *GMR8* glutamate metabotrophic receptor 8; *GRIA3* glutamate receptor ionotrophic alpha-amino-3-hydroxy-5-methyl-4-isoxazole propionic acid 3; *BDNF* brain-derived neurotrophic factor; *DAT1* dopamine active transporter 1; *SLC6A3* solute carrier family 6 transporter member 3; *SLC18A2* vesicular monoamine transporter 2; *MB-COMT* membrane-bound catechol-O-methyltransferase; *S-COMT* soluble catechol-O-methyltransferase; *COMTD1* catechol-O-methyltransferase domain containing 1; *RELN* reelin

Key

Hyper-methylation: ^a^

Hypo-methylation: ^b^

Differential methylation: ^c^

No difference in methylation: ^d^

### Reelin

Differential methylation for *RELN* was reported for intron 1 in SZ [20], although another study also reported a lack of differential methylation of the *RELN* promoter in SZ [22].

### Brain-derived neurotrophic factor

Methylation investigations for *BDNF* in SZ and BD were reported only for promoter regions. In BD (both type I and II) there was consistent reporting of hypermethylation of the *BDNF* exon 1 promoter in two studies [44, 45], although one other study of BD (unspecified-type) reported lack of differential methylation at this site [43]. In SZ, the results were mixed with hypermethylation of *BDNF* promoter I [25], differential methylation of an unspecified *BDNF* promoter [30] and a lack of differential methylation of *BDNF* promoter IV [25].

### Dopamine

There was mixed evidence for methylation status of genes associated with dopamine transporters in SZ, which included hypermethylation of dopamine active transporter 1 (*DAT1*) [30], hypomethylation of solute carrier family 6 transporter member 3 (*SLC6A3*) [37] and differential methylation of vesicular monoamine transporter 2 (*SLC18A2*) [27]. The other inconsistent results for genes associated with dopamine were for *COMT* with studies reporting hypomethylation of membrane-bound *(MB-) COMT* in SZ and BD [52], hypomethylation of *COMT* (isoform not specified) in SZ (but not BD) [51], hypermethylation of soluble *(S-) COMT* in SZ [34], differential methylation of *COMT* domain containing 1 (*COMTD1*) promoter in SZ [37] and a lack of differential methylation of *S-COMT* promoter in SZ [36]. One global DNA methylation study also reported differential methylation of glial cell line-derived neurotrophic factor family receptor alpha 2 *(GFRA2)* in SZ [28], which indirectly affects dopaminergic neurons.

### Serotonin

The reported results for serotonin were varied: hypermethylation of 5-hydroxytryptamine serotonin 1A receptor *(5-HTR1A)* in SZ and BD in two studies [49, 51], a lack of differential methylation of 5-hydroxytryptamine serotonin 2A receptor *(HTR2A)* in SZ and BD [50], differential methylation of serotonin 2A receptor (*SLC6A4*) in BD [48], differential methylation of 5-hydroxytryptamine serotonin receptor 1E G protein-coupled (*HTR1E*) in SZ [37] and a lack of differential methylation of serotonin neurotransmitter transporter (*5-HTT)* in SZ [34].

### Glutamate

Methylation of glutamatergic receptors were reported only in SZ participants; in two studies, there was differential methylation of the glutamate receptor ionotrophic alpha-amino-3-hydroxy-5-methyl-4-isoxazole propionic acid 2 (*GRIA2*) [21] and hypermethylation of glutamate receptor ionotrophic alpha-amino-3-hydroxy-5-methyl-4-isoxazole propionic acid 3 (*GRIA3*) and glutamate metabotrophic receptors 2, 5 and 8 (*GMR2, GMR5* and *GMR8*) [29].

### Subgroup analyses

Subgroup analyses and/or the study of covariates were reported in 28 out of 33 studies. However, only 20 studies reported significant effects of age, sex, pharmacological (antipsychotic/antidepressant) treatment, symptom severity, and/or smoking/alcohol abuse. Further analyses of ethnicity effects on DNA methylation was absent in all but one study [33]. Other notable findings, which were only reported in one study, include a significant association of gene expression with DNA methylation in SZ [41], and cellular heterogeneity of white blood cells as a major confounder in DNA methylation analyses also in SZ [26].

## Discussion

This review highlights findings of moderate quality, showing mixed evidence of hyper- and hypomethylation of several common genetic loci in 22 studies of schizophrenia and/or bipolar disorder, from a total of 33 reviewed studies. Differential methylation converged on five candidate genes (*RELN*, *BDNF*, *COMT, 5-HTT* and glutamate receptor genes) which have each been previously implicated in the neuropathology of SZ and/or BD. Differential methylation was also reported in several genes (e.g. Fc fragment of IgA *(FCAR)*, cyclic AMP-responsive element-binding protein 1 *(CREB1)*, lymphocyte transmembrane adaptor 1 *(LAX1*)) related to immune system function and the inflammatory response in SZ [20, 33], consistent with recent evidence for shared genetic risk (for SZ and BD) in common variants of the major histocompatibility complex [53].

### Genes implicated in schizophrenia and bipolar disorder

The most commonly reported sites of epigenetic changes were in regions known to regulate the availability of neurotrophins, dopamine and serotonin. For example, BDNF is a neurotrophin involved in neuroplasticity and dopaminergic neuron survival [54], for which peripheral blood levels have been found to be decreased in both SZ and BD patients [55, 56]. However, in BD, there were two studies reporting hypermethylation of the *BDNF* gene (exon 1 promoter) that was associated with pharmaceutical treatment and mood states [44, 45], while another study reported a lack of differential methylation at this site in BD patients with a history of psychosis [43]. In SZ, there was evidence of differential methylation at several other *BDNF* sites, including promoter I [25], and an unspecified *BDNF* promoter [30], with one study also reporting no difference in methylation of *BDNF* promoter IV [25].

On the *COMT* gene, there was consistent evidence for hypomethylation of *MB-COMT* in SZ and BD [52] and an unspecified *COMT* isoform in SZ only [51]; other SZ studies reported mixed findings including hypermethylation of *S-COMT* [34], differential methylation of *COMTD1* promoter [37] and a lack of significant differential methylation of *S-COMT* promoter [36]. The mixed evidence for methylation of dopamine transporter genes in SZ – including hypermethylation of *DAT1* [30], hypomethylation of *SCL6A3* [37] and differential methylation of *SLC18A2* [27], is interesting in the context of previous evidence of genome-wide differential methylation of *GFRA2* in SZ [28], a receptor for glial cell-derived neurotrophic factor (GDNF) which manages dopaminergic neuronal maintenance while also being implicated in SZ and BD [57, 58]. Non-specific, differential methylation of serotonin transporter sites were evident in BD (*SLC6A4*) [48] and SZ (*HTR1E*) [37], while hypermethylation of *5-HTR1A* was reported in two SZ and BD studies [49, 51]. These results converge with the numerous reports of variation in serotonin transporter gene (*5-HTT* or *SLC6A4*) interacting with stressful life events to result in psychiatric (usually mood) disorder [59, 60]. However, there was also evidence for lack of differential methylation of *5-HTT* in SZ [34], and *HTR2A* in SZ and BD [50].

Finally, a number of glutamate receptor genes (*GRIA2, GMR2, GMR5, GMR8* and *GRIA3*) were found to be hypermethylated in SZ [21, 29] while in BD there was no such evidence. This is intriguing given that recent genome-wide association studies (GWAS) have implicated genes associated with glutamate neurotransmitter dysfunction as relevant to risk for both disorders [61, 62]. In SZ, there was also a finding of hypermethylation of *DLG4* [33], a gene which has downstream regulatory effects on glutamate receptors implicated in SZ pathophysiology [63]. The few studies of methylation in the promoter region of *RELN* in SZ should be mentioned as consistent with post-mortem evidence [64], while there were some other notable findings for hypermethylation of *DISC1* [32], differential methylation of *HLA-C* and *CALHM1* [41], and hypermethylation of *GABRB2* [39] which have each been identified as risk variants for SZ in previous work [65–67].

### Associations with demographic and clinical variables

Only 20 of 33 studies examined the effects of age, sex, medication, symptom severity, and/or smoking/alcohol abuse on methylation patterns, with mixed findings. However, there were consistent trends emerging for *no* significant associations between methylation status of various genes and age [23, 25, 34, 38, 45, 47, 49] (particularly in SZ studies [23, 25, 34, 38]), while a handful of other studies suggest that differential methylation increases with age [50–52]. There was also a trend for hypermethylation being more prevalent in females (see Tables 1, 2 and 3). Previous studies have reported altered DNA methylation in SZ and BD following treatment with antipsychotics and mood stabilisers such as haloperidol [34], clozapine [68], lithium and valproate [44], but these variables were inconsistently reported in the studies reviewed here.

### Limitations

There are a number of limitations to this review. The most obvious was the inability to conduct a meta-analysis owing to the diversity of experimental protocols (there were 16 different methods reported across 33 studies). Methodological variability also precludes interpretation of results for the most commonly reported genes across these studies. In addition, the lack of consistency in reporting the potential effects of clinical symptoms, age, sex, medication, and ethnicity, precluded adequate interpretation of findings across studies. For example, factors such as diet [69], exercise [70], smoking [71], trauma [72], emotional state [73] and ethnicity [74] are known to effect DNA methylation status, but were not adequately reported in many studies. Variability in DNA extraction methods and blood cell composition may have also affected the results of included studies [35], for which details are not included in this review. For example, the cellular heterogeneity of white blood cells has been considered to confound DNA methylation analyses [26], despite associations between gene expression and DNA methylation in whole blood samples suggesting that differences are minimal. However, methods for conducting methylation analyses are known to vary in efficacy and sensitivity, and may have affected the pattern of results revealed here. For example, bisulfite sequencing (conducted in eight of 33 included studies), is prone to PCR amplification bias [75], with at least some incomplete conversions of cytosine to uracil resulting in a higher number of methylated CpGs being recorded [76]. Moreover, methods for determining the appropriate significance threshold to determine differential methylation status was dependent on the experimental protocol employed in each study, such that the strength of the results reported in these studies may be equivocal. Another potential bias lies in the sample size differences between studies of candidate gene and global DNA methylation studies, of which the latter require larger sample sizes with respect to multiple testing issues. In addition, the results of global DNA methylation studies are simply not comparable with approaches such as MBD protein-enriched genome sequencing which is more sensitive than 27 k/450 k arrays [21].

Finally, this study did not directly compare the methylation status of particular genes arising from studies of post-mortem versus peripheral tissue, but included studies using DNA derived from blood or saliva (only two studies used saliva). While methylation patterns in saliva cells may be affected by oral hygiene, we note that similar patterns of methylation were reported in saliva and post-mortem tissue in both these studies [50, 52]. There are certain advantages and limitations to using both post-mortem and peripheral tissues for DNA methylation studies. While the brain is the primary organ of pathology in SZ and BD, methylation analyses using post-mortem tissue may be affected by pH, post-mortem interval and variability of different neuronal cell types and brain regions [77]. On the other hand, peripheral tissue is easily accessible in a minimally invasive and of low cost procedure, thus allowing for collection of larger sample sizes to overcome cellular heterogeneity of methylation patterns and facilitating longitudinal studies. Consistent methylation results across brain and blood tissues have been reported for particular promoter CpG islands in other studies not included in this review [17]. Notably, two of the included studies showed comparable methylation results for major histocompatibility complex 9 *(HCG9)* [46] and synapsin 3 *(SYNIII)* [35] in both post-mortem brain tissue as well as a blood-derived DNA.

## Conclusions

Moderate quality evidence shows differential DNA methylation in peripheral tissue of SZ and BD participants, with some common genes affected despite the direction of methylation at common sites not always being consistent. While it remains questionable as to whether the differences in statistical thresholds between genome-wide to candidate gene loci studies are too great to compare results from such studies, we could not systematically address the effects of these methodological difference on general patterns of findings across disorders because of the limited data available for some methods in some groups. We also note that the role of DNA methylation in modifying gene expression has only been explored in the last 20 years [78], and it is likely that the inconsistency of the results reported here reflects the numerous methods available for determining DNA methylation alterations in peripheral tissues of these disorders, which can vary according to cell type. Reliable patterns in methylation alterations specific to SZ or BD are likely to emerge with improved, cost-effective and standardised technology that also account for cellular heterogeneity. Apart from methodological issues, another potential explanation for variability among findings is that none of the studies of psychotic samples to date have addressed the impact of early life experiences (such as childhood trauma) on differential methylation patterns in SZ and BD; more consistent methylation patterns may be revealed in the context of common lifetime environmental exposures (e.g., childhood maltreatment, birth complications, cannabis use), genomic structure, and/or mRNA expression profiles. The consideration of these additional factors will be necessary in future research to clarify the contribution of environmental effects on epigenetic processes in the development of psychosis.

## Abbreviations

SZ: Schizophrenia
BD: Bipolar disorder
HC: Healthy control
STROBE: Strengthening the reporting of observational studies in epidemiology
PRISMA: Preferred reporting items for systematic reviews and meta-analyses
MWAS: Methylome-wide association study
GWAS: Genome-wide association study
DNA: Deoxyribonucleic acid
mRNA: Messenger ribonucleic acid
PCR: Polymerase chain reaction
MBD: Methyl-CpG binding domain
MSRE: Methylation sensitive restriction enzyme
HRM: High-resolution melt
HPLC: High-performance liquid chromatography
MB-: Membrane-bound
S-: Soluble
GNDF: Glial cell-derived neurotrophic factor
